# Blanking period antiarrhythmic drugs after catheter ablation for atrial fibrillation: a meta-analysis of randomized controlled trials

**DOI:** 10.3389/fcvm.2023.1071950

**Published:** 2023-07-20

**Authors:** Gang Chen, Guangling Li, Demei Zhang, Xiaomei Wang, Xueya Guo

**Affiliations:** ^1^ Department of Cardiology, Lanzhou University Second Hospital, Lanzhou University, Lanzhou, China; ^2^ Department of Cardiology, Lanzhou University Second Hospital, The Second Clinical Medical College of Lanzhou University, Lanzhou, China

**Keywords:** atrial fibrillation, antiarrhythmics, clinical trials, catheter ablation, meta-analysis

## Abstract

**Background:**

Antiarrhythmic drugs (AADs) are frequently prescribed following catheter ablation (CA) for atrial fibrillation (AF). However, to date, there is a lack of large-scale, multicenter controlled studies that have confirmed the efficacy of AADs in reducing the incidence of late recurrence of AF after CA. Furthermore, the optimal duration of short-term use of AADs after CA remains a controversial topic.

**Methods:**

PubMed, Embase, Cochrane Library, CNKI, and ClinicalTrials.gov were searched until April 25, 2022. We conducted a meta-analysis of randomized controlled trials (RCTs) to assess the efficacy of blanking period AADs in predicting both early and late recurrence of AF. In addition, Grading of Recommendations Assessment, Development and Evaluation (GRADE) was used to assess the quality of evidence in this meta-analysis.

**Results:**

12 RCTs with 3,625 patients were included in this meta-analysis. Short-term use of AADs after AF ablation reduced the risk of early recurrence of AF compared with the no-AADs group. In the subgroup analysis of AADs use time, it was found that only using AADs for more than 2 months can reduce the early recurrence of AF after CA. However, when compared with the no-AADs group, short-term use of AADs after CA did not reduce the incidence of late recurrence of AF.

**Conclusions:**

Short-term use of AADs (more than 2 months) can reduce the early recurrence but not the late recurrence of AF after CA.

## Introduction

Atrial fibrillation (AF) is a common arrhythmia in clinical practice that increases the risk of stroke and heart failure. As of 2019, there were approximately 59.7 million patients of AF (including atrial flutter) worldwide (1). While the lifetime risk of AF was previously about one in four (2, 3), recent studies have reported that one in three people of European ancestry over 55 has AF (4, 5). The EAST-AFNET4 study showed that in patients with newly diagnosed AF within 1 year, the incidence of major cardiovascular events in early rhythm control was lower than that in the conventional treatment group (mainly ventricular rate control). Additionally, the maintenance of sinus rhythm is higher in the early rhythm control group. Catheter ablation (CA) can be used as the first treatment for AF. However, due to proarrhythmic milieu caused by CA lesions, AF recurrence is common within the first few months of post-ablation. Because of “AF begets AF”, those patients with early recurrence after post-ablation are also more likely to have a late recurrence (6). This notion has also been confirmed that post-ablation blanking period episodes are independent predictors of AF recurrence (7). Therefore, the use of AADs after AF ablation aim not only to reduce early recurrence but also to reduce late recurrence. However, most of the existing studies have shown that the use of AADs cannot reduce the late recurrence of AF after CA. It should be noted that the sample size of previous studies is not enough to clarify this issue, so this conclusion is controversial.

Xu et al. (8) conducted a meta-analysis to evaluate the efficacy of AADs and found that short-term use of AADs could reduce the incidence of early recurrence of AF but could not prevent late recurrence of AF. However, the study had several limitations. First, this meta-analysis included few studies, only six randomized controlled trials (RCTs), which resulted in an insufficient sample size. Second, sources of heterogeneity were not adequately analyzed (Subgroup analysis was not sufficient and meta regressions were not performed). Third, exploration of the stability of the results is not enough (Subgroup analysis was not sufficient and sensitivity analysis was not performed). Finally, an assessment of publication bias was not performed. In addition, they did not evaluate a more meaningful indicator: the use time of AADs in the blanking period after CA of AF, which is crucial for clinical treatment. To shed further light on this issue, we conducted a meta-analysis that, in addition to ameliorating the deficits mentioned above, explored the duration of drug use to reduce AF early recurrence after CA. Simultaneously, we evaluated the quality of evidence using the Grading of Recommendations Assessment, Development and Evaluation (GRADE) approach to facilitate its clinical application.

## Methods

This meta-analysis was performed according to the recommendations of the Cochrane Handbook for Systematic Reviews of Interventions and The Preferred Reporting Items for Systematic Reviews and Meta-Analysis (PRISMA) checklist guided the protocol reporting (9). There was no registered protocol for this meta-analysis.

### Literature search strategy

PubMed, Embase, Cochrane Library, CNKI, and ClinicalTrials.gov were searched until April 25, 2022. The systematic electronic searches were conducted using exploded Medical Subject Headings (MeSH) terms and the corresponding keywords in Title, Abstract, or All Fields. The search terms used in this meta-analysis were (MeSH exp “Atrial Fibrillation”, and keywords “atrial fibrillation OR atrial fibrillat* OR auricular fibrillat* OR atrium fibrillat* OR AF”), (MeSH exp “AntiArrhythmia Agents”, and keywords “Antiarrhythmia agents OR anti-arrhythmia drugs OR antiarrhythmi* OR procainamide OR disopyramide OR mexiletine OR flecainide OR propafenone OR bisoprolol OR esmolol OR amiodarone OR dofetilide OR sotalol OR ibutilide OR azimilide OR moricizine OR cibenzoline”), and (MeSH exp “Catheter Ablation” and keywords “catheter ablation OR radiofrequency OR cryoablation OR PVI OR pulmonary vein isolation”). We applied filters to restrict the type of trials to RCTs involving human subjects only, without any language restrictions. To ensure that no relevant articles were overlooked, we conducted a subsequent search on April 28, 2022. In addition, we manually searched the references in the included literature to identify potential eligible trials.

### Selection criteria

Published studies meeting the following criteria were included: (1) Patients: AF patients underwent CA with pulmonary vein isolation (PVI)-based strategy; (2) Intervention: patients were treated with AADs within the first 3 months after CA (blanking period); (3) Comparison: patients were not on AADs treatment, either placebo or usual care; (4) Outcome: early recurrence of atrial arrhythmia lasted more than 30 s within the first 3 months after CA, and late recurrence of atrial arrhythmia lasted more than 30 s post the 3 months after CA; and (5) Study type: all articles included were RCTs. Exclusion criteria: (1) We excluded duplicate reports; (2) We excluded conference abstracts unless they were accompanied by a full-text publication in a peer-reviewed journal; (3) We excluded animal experiments; (4) We excluded performed additional atrioventricular node ablation, pacing therapy, and surgical ablation patients.

### Study inclusion and data extraction

Two reviewers (G.L. and G.C.) conducted initial searches, removed duplicates, and screened titles and abstracts to identify eligible articles. In instances where there were discrepancies in the inclusion of literature, the full-text article was obtained to determine eligibility. Any uncertainties or disagreements were resolved through discussion and consensus. Data collection was performed by G.L. and independently confirmed by other authors (G.C. and D.Z.). Additionally, we also reviewed supplementary appendices of included RCTs and contacted the corresponding authors to verify extracted data and request the unavailable data, if needed. All discrepancies were resolved by discussion and consensus. The scheduled primary outcome was the early recurrence, and the secondary outcome was the late recurrence of AF.

### Risk of bias assessment and grading quality of evidence

The risk of bias was assessed in duplicate by independent reviewers (G.L. and G.C.) using the Cochrane risk-of-bias tool (10). A study was considered high risk if only one domain of the trials had rated as high risk; the study was regarded as low risk if all domains had rated as low risk; otherwise, they were considered at unclear risk of bias. The quality of the evidence in this meta-analysis was independently assessed (G.L. and G.C.) according to the GRADE.

### Statistical analysis

Statistical analyses were performed using Stata 14.0 and Review Manager 5.4. Relative risk (RR) was used as the effect size indicator for enumeration data. Point estimates and 95% confidence interval (CI) were calculated for dichotomous outcomes. The heterogeneity of the included studies was analyzed using the *Q* test (test level *α *= 0.1), and the *I*^2^ statistic was used to quantify the heterogeneity between studies. *I*^2^ values between 25% and 50% were considered mild heterogeneity, between 50% and 75% were considered moderate heterogeneity, and those above 75% were considered high heterogeneity (11). Regardless of the value of *I*^2^, the Mantel-Haenszel method was used to pool the RR and 95% CI with the random-effects model. If there was significant heterogeneity in this meta-analysis, subgroup analyses are applied to identify sources of heterogeneity. Prespecified subgroup analyses included time of drug use, publication time of literature, the sample size of individual studies, and the risk of individual studies. In addition, sensitivity analysis was applied to assess the effect of individual or small sample studies on the overall effect size. If the heterogeneity was significant and could not be resolved by the above methods, the meta-analysis was abandoned, and only qualitative analysis was performed. Since the visual assessment of whether a funnel plot was symmetric could be subjective, we used the Egger test to detect potential publication bias (12). *P*-value < 0.05 was considered statistically significant unless previously defined, such as *p*-value < 0.1 indicated statistically significant for heterogeneity test.

## Results

### Literature search

**Figure 1:**
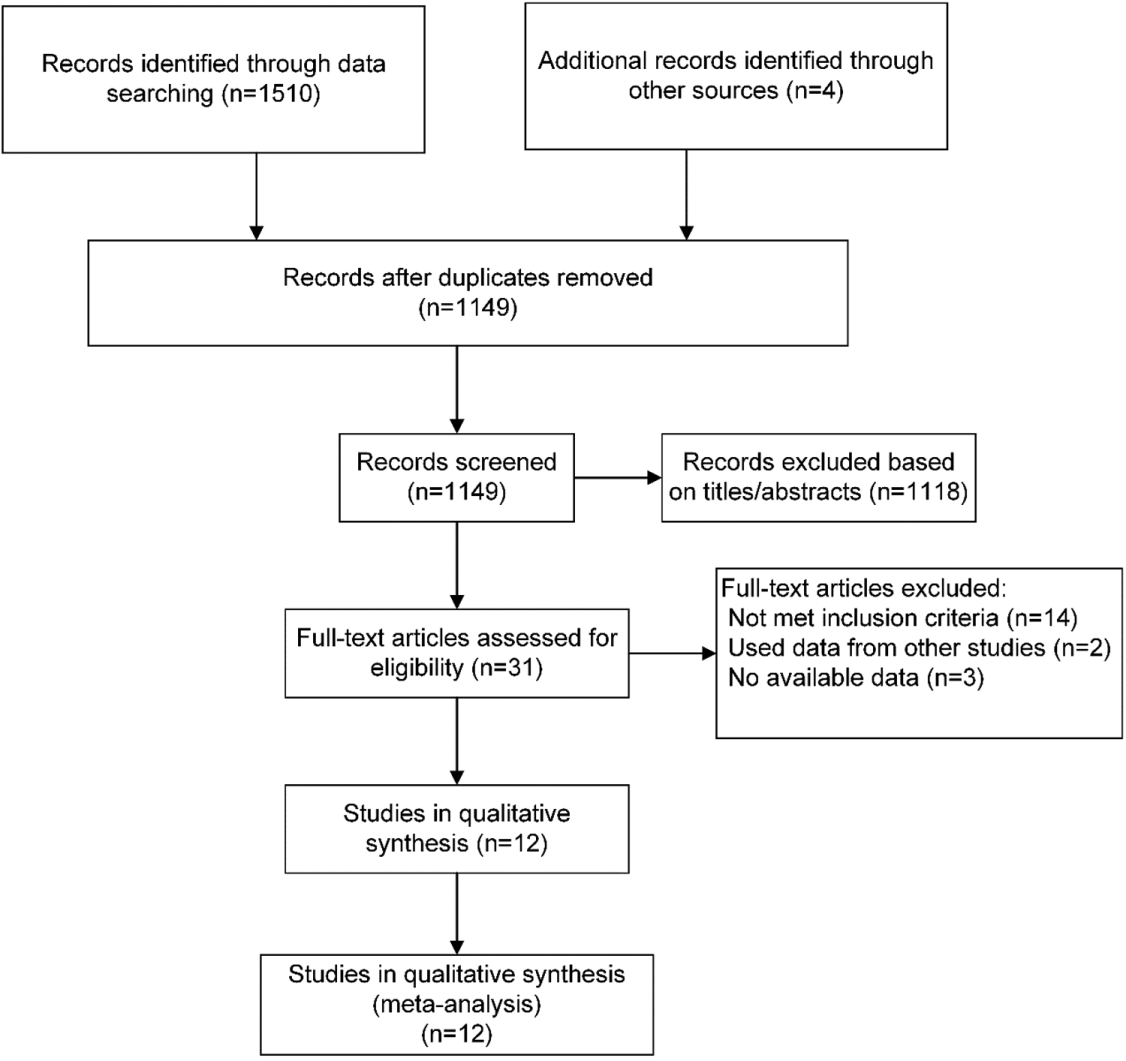
Selection of RCTs for this meta-analysis.

The literature search and selection results are shown in the PRISMA flowchart (Figure 1). Our initial search yielded 1,510 articles. After removing duplicates and screening titles and abstracts, 31 articles were considered likely to meet the inclusion criteria. After a full-text review, 12 published articles from 11 RCTs were finally included in this meta-analysis. Two articles were derived from the same study with different results at 6 weeks and 6 months.

### Trials characteristics and risk of bias assessment

**Figure 2:**
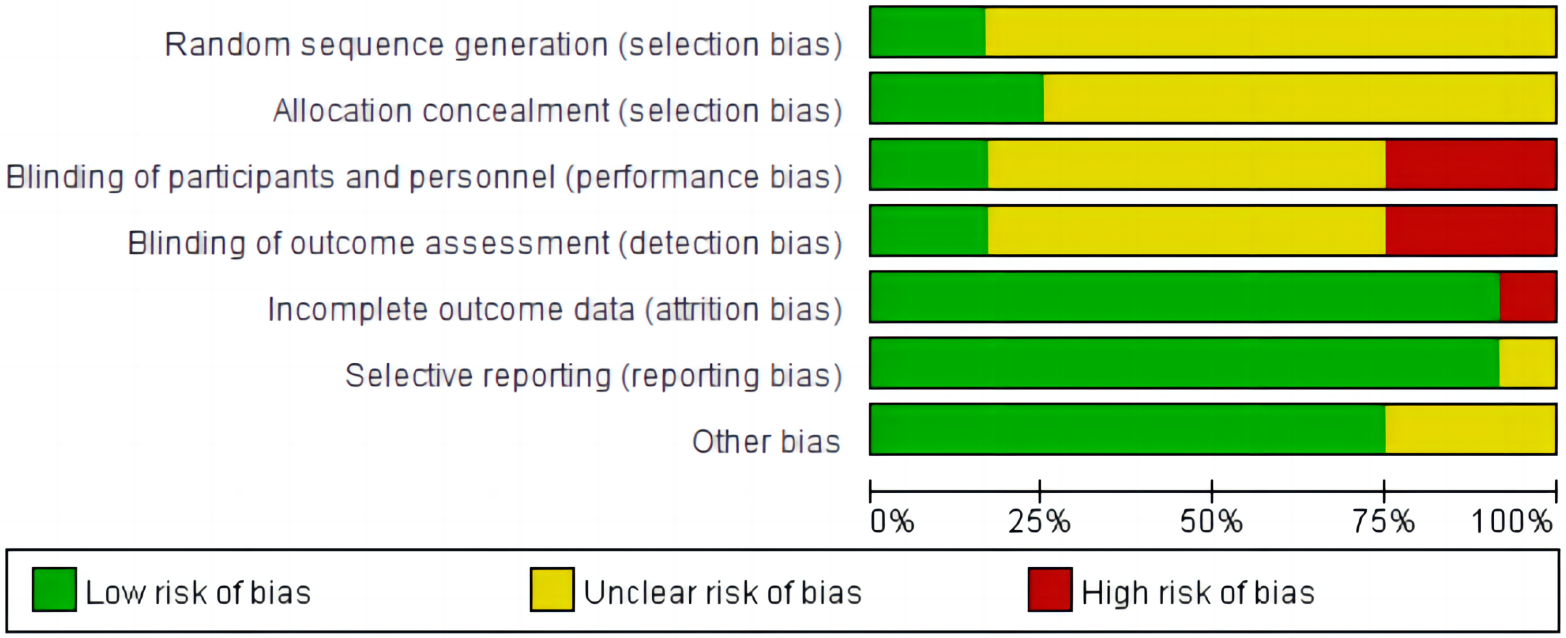
Risk of bias for this meta-analysis: judgments about each risk of bias item presented as percentages across all included randomized controlled trials.

**Figure 3:**
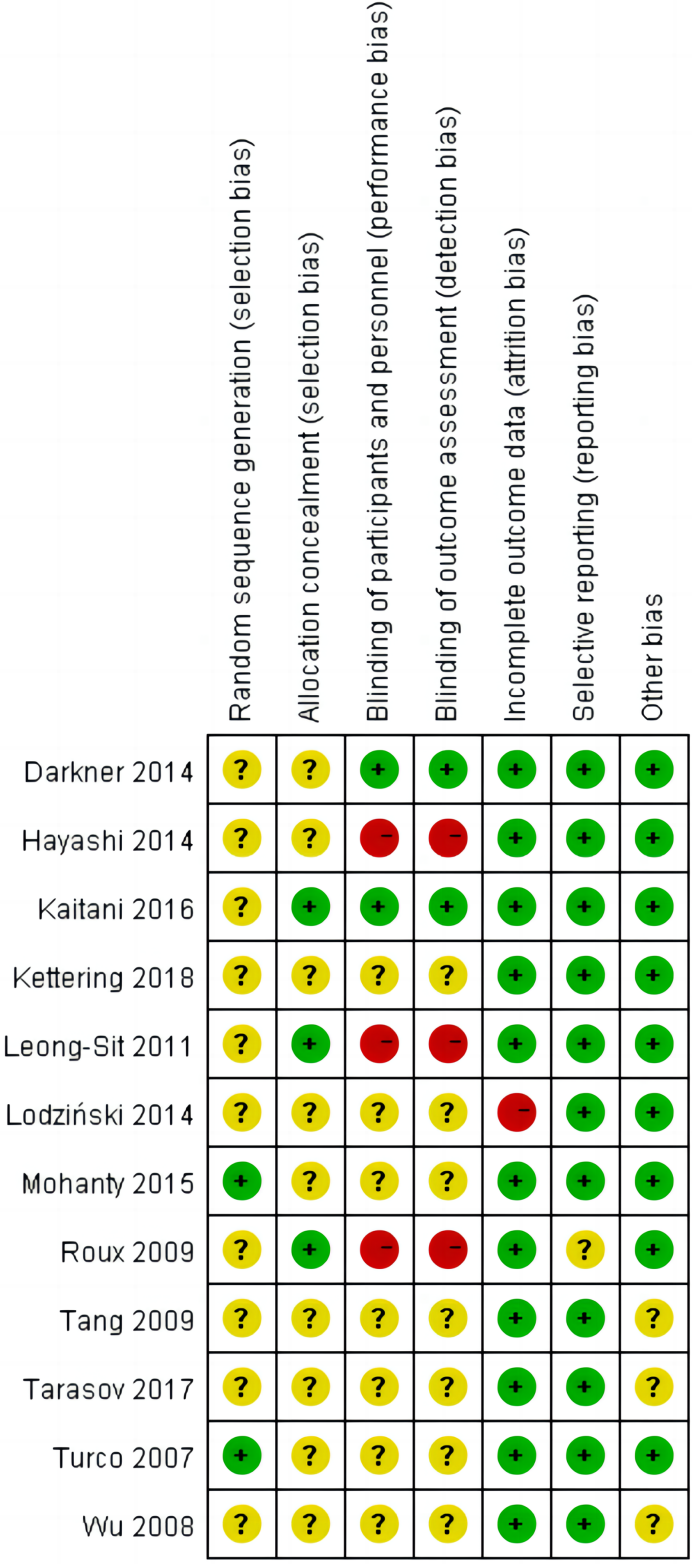
Summary of risk of bias summary of the included randomized controlled trials: details about each risk of bias item for each included trial. Green = low risk of bias, Yellow = unclear risk of bias, Red = high risk of bias.

The main characteristics of included 12 RCTs with 3,625 patients are shown in Table 1. The sample size of trials ranged from 74 to 2,038. A total of 1,937 patients were administered AADs, while 1,689 patients received no-AADs treatment. The primary AAD used was amiodarone. All patients underwent CA using a PVI-based strategy. Patients in the AAD group received drug treatment lasting between 6 weeks and 3 months. The RCTs had a mean follow-up duration ranging from 3 to 28 months. Detailed information on the risk of bias is presented in Figures 2, 3.

**Table 1:** Included studies: key characteristics and monitoring details.

Author (year)	Study location	AF pattern	Sample size	Male (%)	Mean age or median (years)	Mean follow-up (months)
Wu et al. (2008) (13)	China	Paroxysmal or persistent	74	77	52.1 ± 18.1	20.5
Turco et al. (2007) (14)	Italy	Paroxysmal or persistent	106	65	57.0 ± 10.0	12.0
Roux et al. (2009) (15)	the United States	paroxysmal	110	71	55.0 ± 9.0	1.5
Kettering and Gramley (2018) (16)	Germany	persistent	230	56	61.2 ± 11.2	24.0
Darkner et al. (2014) (17)	Denmark	Paroxysmal or persistent	212	83	61.0, IQR (54.0–66.0)	6.0
Leong-Sit et al. (2011) (7)	the United States	Paroxysmal	110	71	55.0 ± 9.0	6.0
Hayashi et al. (2014) (18)	Japan	Paroxysmal or persistent	125	77	63.0 ± 11.0	17.0
Lodziński et al. (2014) (19)	Poland	No description	171	72	50.2 ± 11.2	2.0
Kaitani et al. (2016) (20)	Japan	Paroxysmal or persistent or long-lasting	2,038	75	63.3 ± 10.0	15.0
Tarasov et al. (2017) (21)	Russian	No description	243	58	56.1 ± 10.1	12.0
Mohanty et al. (2015) (22)	Multi-center	long-lasting	106	71	61.0 ± 10.5	32.0
Tang et al. (2009) (23)	China	Paroxysmal or persistent	210	70	57.0 ± 12.0	6.0
Author (year)	Class of AADs	AADs period (month)	Monitoring for recurrence		Definition of recurrence	
Wu et al. (2008) (13)	Amiodarone	3.0	Ambulatory ECG once a month; ECG when experiencing symptoms.		Any documented ATa lasting ≥30 s Early: within 3 months Late: >3 months	
Turco et al. (2007) (14)	Class Ⅰ C or Amiodarone	1.0	Weekly transtelephonic ECG; ECG and ambulatory ECG at month 1, 4, 7, 10 and 13 visits.		Any documented ATa lasting ≥30 s Early: within 1 month Late: >1 month	
Roux et al. (2009) (15)	Class Ⅰ C or Class Ⅲ	1.5	Auto-trigger transtelephonic monitor for 30 days.		Any documented ATa lasting ≥24 h Early: within 6 weeks Late: no definition	
Kettering and Gramley (2018) (16)	Amiodarone	3.0	ECG and ambulatory ECG at month 1 and 3 visit, then every 3 months; after 1 year, every 12 months.		Any documented ATa lasting ≥30 s Early: within 3 months Late: >3 months	
Darkner et al. (2014) (17)	Amiodarone	2.0	ECG at month 1, 3 and 6 visits; ambulatory ECG at 6 weeks and 6 months.		Any documented ATa lasting ≥30 s Early: within 3 months Late: >3 months	
Leong-Sit et al. (2011) (7)	Class Ⅰ C or Class Ⅲ	1.5	30-day transtelephonic monitor at post-operative and month 6.		Early: within 6 months, any documented ATa lasting ≥24 h Late: >3 months, any documented AF lasting ≥1 min	
Hayashi et al. (2014) (18)	Flecainide	3.0	Cardiac event recorder for 4 months twice a day for 30 s each; ECG at week 2, month 1, 2, 3, 4, 5, 6, 8, 10, and 12 visits, then every 3 months; ambulatory ECG at month 6 and 12 visits.		Any documented ATa lasting ≥30 s Early: within 3 months Late: >3 months	
Lodziński et al. (2014) (19)	Class Ⅰ C or Class Ⅲ	2.0	24-h Holter ECG at month 1 and 2 visits.		Any documented ATa lasting ≥30 s Early: within 2 months Late: no definition	
Kaitani et al. (2016) (20)	Class Ⅰ or Class Ⅲ	3.0	ECG at month 3, 6, and 12 visits; ambulatory ECG at month 6 and 12.		Any documented ATa lasting ≥30 s Early: within 3 months Late: >3 months	
Tarasov et al. (2017) (21)	Class Ⅰ C or Class Ⅲ	3.0	ECG, Holter monitor, and 112 patients had an implantable loop recorder for constant monitoring.		Based on PC and EC Early: within 3 months Late: no definition	
Mohanty et al. (2015) (22)	Amiodarone	2.0	ECG and Holter ECG at month 3, 6, 9, 12 visits, then every 6 months; event recorder for 5 months after ablation at least twice per week.		Any documented ATa lasting ≥30 s Early: within 2 months Late: >2 months	
Tang et al. (2009) (23)	Class Ⅰ C or Class Ⅲ	3.0	ECG and Holter ECG at month 1, 3, 6, 12 visits, then every 6 months.		Any documented ATa lasting ≥30 s Early: within 3 months Late: >3 months	

AAD, antiarrhythmic drug; AF, atrial fibrillation; ATa, atrial tachyarrhythmia (atrial fibrillation, atrial tachycardia, atrial flutter); ECG, electrocardiography; PC, pharmacological cardioversions; EC, electrical cardioversions.

### Primary outcomes

**Figure 4:**
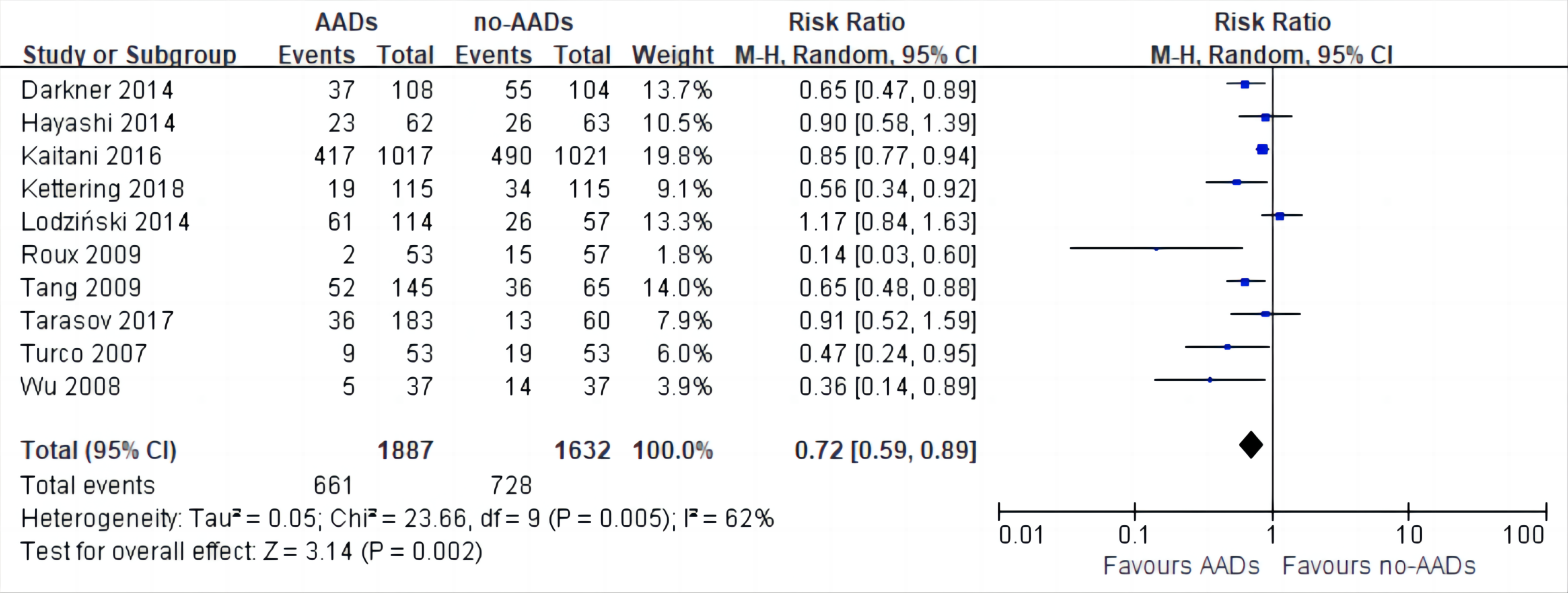
Effect of short-term AADs use versus no-AADs prescription after atrial fibrillation ablation on early recurrence of atrial arrhythmias.

**Figure 5:**
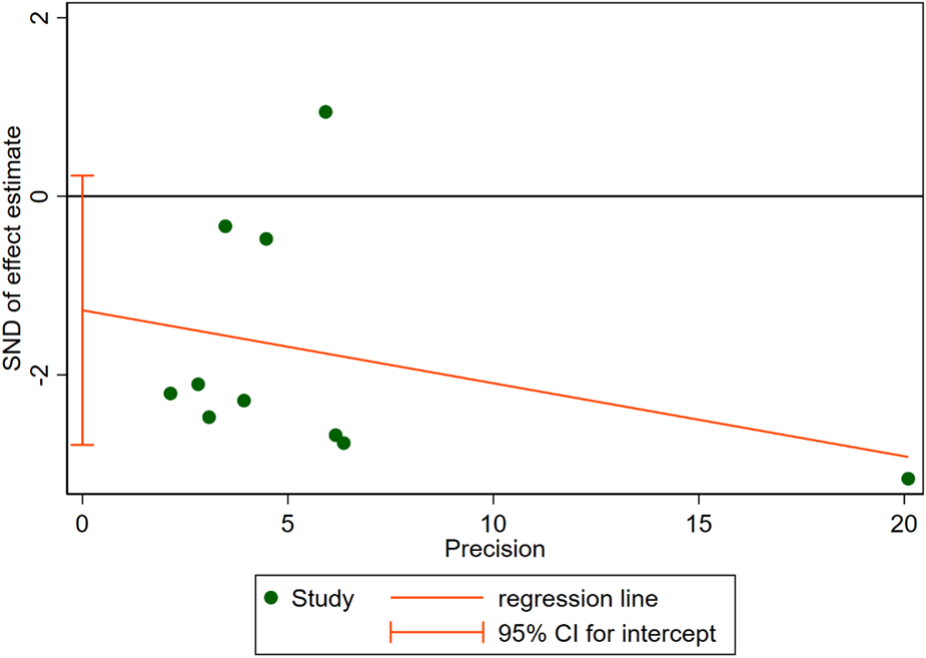
Egger test for publication bias in the meta-analysis.

10 RCTs including a total of 3,519 patients were analyzed to provide relevant evidence regarding the primary outcomes of early recurrence of AF. Short-term use of AADs significantly reduced the risk of AF recurrence after CA in the blanking period compared to the no-AADs prescription group (RR, 0.72; 95% CI, 0.59–0.89; *p* = 0.002; Figure 4), with moderate heterogeneity (*I*^2^ = 62%; *p*_het_ = 0.005). Given the moderate heterogeneity, subgroup analysis was performed to evaluate the influence of different groups on primary outcomes. When grouped by publication time <2014 and ≥2014, it can be seen that the *I*^2^ of both groups decreased to less than 50%, and publication time can be considered the main source of heterogeneity in this meta-analysis (Supplementary Figure S1). In addition, when grouped by drug use time, it can be found that drug use time ≤2 months is ineffective (RR, 0.67; 95% CI, 0.43–1.06; *p* = 0.09) for early recurrence of AF, while drug use time >2 months is effective (RR, 0.75; 95% CI, 0.62–0.91; *p* = 0.004; Supplementary Figure S2). Further, when grouped by sample size and risk of study, respectively, it can be found that the subgroup with a sample size of less than 200 and the subgroup with a high risk of study both obtained the same conclusion that the use of AADs in the blanking period is ineffective for the early recurrence of AF. The subgroup with a sample size of more than 200 and the subgroup with no-high risk of study obtained the opposite conclusion (Supplementary Figures S3, S4). To assess the robustness of this meta-analysis, a sensitivity analysis consecutively excluding one trial each time was applied to assess the impact of individual studies. As a result, no trials had significant effect on the pooled estimate and 95% CI (Supplementary Figure S5). We conducted an assessment of publication bias using the Egger test (Figure 5) and found no evidence of potential publication bias.

### Secondary outcomes

For late recurrence of AF, 2,929 patients were included in nine RCTs. Compared with no-AADs prescription group, short-term use of AADs did not reduce the risk of late recurrence of AF after CA (RR, 0.96; 95% CI, 0.88–1.06; *p* = 0.45; Supplementary Figure S6), with mild heterogeneity (*I*^2^ = 29%; *p*_het_ = 0.19). Although such heterogeneity is acceptable, we further evaluated the stability of this meta-analysis. Similar methods were used to evaluate the late recurrence of AF. We performed the same subgroup and sensitivity analyses in the late recurrence of AF. All results showed that short-term use of AADs cannot reduce the risk of late recurrence of AF (Supplementary Figures S7–S11).

### GRADE profile evidence

The GRADE profile evidence for this meta-analysis in the primary and secondary outcomes is presented in Table 2.

**Table 2:** The GRADE evidence profile for the primary and secondary outcomes of this meta-analysis.

Quality assessment							No. of patients		Effect		Quality	Importance
No. of studies	Study design	Risk of bias	Inconsistency	Indirectness	Imprecision	Other considerations	Short-term AADs	Control	Relative (95% CI)	Absolute (95% CI)		
Early recurrence of AF after CA following short-term AADs use												
10	RCTs	Serious^a^	No serious^b^	No serious	No serious	None	669/1,887 (35.5%)	737/1,632 (45.2%)	RR 0.73 (0.61–0.88)	122 fewer per 1,000 (from 176 fewer to 54 fewer)	⨁⨁⨁◯ Moderate	CRITICAL
Late recurrence of AF after CA following short-term AADs use												
9	RCTs	Serious^c^	No serious	No serious	No serious	None	532/1,500 (35.5%)	531/1,429 (37.2%)	RR 0.96 (0.88–1.06)	15 fewer per 1,000 (from 45 fewer to 22 more)	⨁⨁⨁◯ Moderate	CRITICAL

RCTs, randomized controlled trials; AF, atrial fibrillation; CA, catheter ablation; CI, confidence interval; RR, risk ratio.

^a^ Although two included RCTs were judged as high risk of performance bias because of without blinding of participants and personnel, and high risk of detection bias because of without blinding of outcome assessment, and one included RCT was judged as high risk of attrition bias because of with incomplete outcome data, the predefined objective outcome was just partly influenced.

^b^ Although *I*^2^ was 62%, grouping by publication time could explain the heterogeneity of this result.

^c^ Although two included RCTs were judged as high risk of performance bias because of without blinding of participants and personnel, and high risk of detection bias because of without blinding of outcome assessment, the predefined objective outcome was just partly influenced.

## Discussion

The main innovations of this meta-analysis are as follows: (1) the topic of this meta-analysis is an essential issue in clinical practice, but it is controversial whether to use AADs in the blanking period after the CA of AF. At the same time, there is insufficient evidence for guidelines recommending the use of AADs in the blanking period. (2) The meta-analysis on this topic is not updated after 2016. Only six RCTs were included in the studies by Xu et al. (8) and Chen et al. (24), the numbers of studies were too small, and there was no way to evaluate publication bias while we addressed this issue. In addition, we adequately evaluated the stability and heterogeneity in this meta-analysis. (3) The previous guidelines and expert consensus did not recommend the specific time of AADs use during the blanking period. Our study demonstrated that even preventing early recurrence of AF requires at least 2 months of AADs use, which helps guide clinical practice.

The main conclusions of this meta-analysis are as follows: (1) The use of AADs in the blanking period can reduce the early recurrence of AF after CA. (2) The use of AADs in the blanking period cannot reduce the late recurrence of AF after CA. Existing studies have shown that 60% of patients with early recurrence automatically return to sinus rhythm, but early recurrence is an independent predictor of AF late recurrence after CA (7, 20, 25). There are two issues that need to be addressed. First, why do patients with early recurrence automatically return to sinus rhythm? Second, AADs can reduce the early recurrence, so they can theoretically reduce the AF late recurrence. However, our study is consistent with previous studies showing that using AADs in the blanking period does not reduce the risk of late recurrence of AF. So how do we interpret this result? Regarding the first issue, there may be the following reasons: (1) Inflammatory response is one of the reasons for the recurrence of AF in the blanking period post-ablation, which is also confirmed by using anti-inflammatory drugs to reduce the early recurrence of AF. In addition, elevated inflammatory cytokines return to baseline within 30 days after CA (26–30). (2) The potential side effect of AF ablation is the imbalance of cardiac autonomic nerve regulation. Studies have found that circumferential pulmonary vein ablation can change the autonomic nerve activity dominating the sinoatrial node, leading to the recurrence of AF, which often disappears within 1 month (31). (3) The electrical conduction of the left atrium and pulmonary veins recover early after CA, which is caused by incomplete ablation lesions. (4) Development of a permanent atrial lesions by scar rigidification may cause delayed cure. In this process, due to the postoperative inflammatory response and autonomic imbalance disappear within 1 month, patients with AF recurrence in the second and third months after CA may be stronger predictors of AF late recurrence than those in the first month. This inference has also been confirmed in some studies (32–34).

As far as the second issue is concerned, the following reasons can partially explain this phenomenon: (1) The most important reason is incomplete PVI, which leads to the restoration of conduction between the left atrium and the pulmonary veins (35, 36); (2) Another common reason is the trigger foci outside the pulmonary veins, which can be mapped and identified after intravenous infusion of high-dose isoproterenol; (3) The experience of CA for AF patients also affects the late recurrence rate of AF. The above problems cannot be effectively solved by the use of AADs.

In addition, we also need to pay attention to the fact that the type of AF and the means of recurrence screening also have an important impact on the late recurrence rate of AF (37, 38). To further exclude the above factors affecting our results, the type of AF and the means of recurrence screening detail are described in our baseline data.

In this meta-analysis, there was moderate heterogeneity (*I*^2 ^= 62%) for the pooled primary outcome, and further subgroup analysis revealed that the main source of heterogeneity was publication time. After grouping by 2014 as the cutoff value, the heterogeneity of the pooled results was significantly reduced. This result can be explained because there has been some progress in CA of AF during this period. Cardiologists came to recognize that the extent of CA lesions depends on the stability of the catheter, contact pressure, energy output, temperature, and ablation time (39). In addition, it is more important to recognize that the initial pulmonary veins isolation is observed for 20–30 min and to reevaluate that a bidirectional block can improve the permanent isolation rate. Some special patients can be monitored for 60–90 min to perform a reevaluation (40).

When we grouped studies by sample size and risk of bias, respectively, we found that studies with a sample size of less than 200 and studies classified as high risk all concluded that AADs were ineffective in preventing early AF relapse. Meanwhile, studies with a sample size greater than 200 and those classified as non-high risk reached opposite conclusion. This suggests that the relatively small sample size and poor quality of the studies may lead to erroneous conclusions. In fact, the result should be more robust. Furthermore, we found that pooled results heterogeneity was low for secondary outcomes, and the results were robust under all grouping factors.

Another notable finding is that previous studies have shown that continuous AADs use for less than 2 months after CA can reduce incidence of atrial arrhythmias during the blanking period. However, our study confirmed that only continuous application of AADs for more than 2 months could reduce the episodes of the blanking period with a larger sample size. This finding provides valuable insights for clinicians regarding the optimal duration of medication during the blanking period.

## Limitations

(1) This meta-analysis does not utilize individual patient data, which limits our ability to identify the presence or absence of effect modification. (2) Many studies included in our meta-analysis were open-label and required additional ECG and/or Holter monitoring if patients developed arrhythmia-related symptoms. Patients in the control group with more ECG and/or Holter monitoring (more likely to experience arrhythmia-related symptoms) had more opportunities to document AF recurrence. (3) The RCTs included in this meta-analysis exhibited differences in terms of the ablation procedures applied, follow-up periods used, physician experience, and type of AADs administered.

## Conclusions

Short-term use of AADs post-AF ablation can lower the risk of early recurrence of AF. However, it does not result in a corresponding reduction in the risk of late recurrence of AF. More importantly, the risk of early relapse can be reduced only when AADs are used continuously for 2 months or more in the blanking period.

## Data Availability

The original contributions presented in the study are included in the article/**Supplementary Material**, further inquiries can be directed to the corresponding author.

## Supplementary material

Supplementary Figure S1

Subgroup analysis of early recurrence of atrial fibrillation after catheter ablation by publication time.

Supplementary Figure S2

Subgroup analysis of early recurrence of atrial fibrillation after catheter ablation by antiarrhythmic drugs (AADs) use time.

Supplementary Figure S3

Subgroup analysis of early recurrence of atrial fibrillation after catheter ablation by sample size.

Supplementary Figure S4

Subgroup analysis of early recurrence of atrial fibrillation after catheter ablation by risk of study.

Supplementary Figure S5

Sensitivity analysis of early recurrence of atrial fibrillation.

Supplementary Figure S6

Effect of short-term antiarrhythmic drugs (AADs) use versus no-AADs prescription after atrial fibrillation ablation on late recurrence of atrial arrhythmias.

Supplementary Figure S7

Subgroup analysis of late recurrence of atrial fibrillation after catheter ablation by publication time.

Supplementary Figure S8

Subgroup analysis of late recurrence of atrial fibrillation after catheter ablation by antiarrhythmic drugs (AADs).

Supplementary Figure S9

Subgroup analysis of late recurrence of atrial fibrillation after catheter ablation by sample size.

Supplementary Figure S10

Subgroup analysis of late recurrence of atrial fibrillation after catheter ablation by risk of study.

Supplementary Figure S11

Sensitivity analysis of late recurrence of atrial fibrillation.
