# Supplementary figures and images for: Blanking period antiarrhythmic drugs after catheter ablation for atrial fibrillation: a meta-analysis of randomized controlled trials
Source: Front Cardiovasc Med. 2023 Jul 20;10:1071950. doi: 10.3389/fcvm.2023.1071950 (PMC10397410; doi:10.3389/fcvm.2023.1071950)

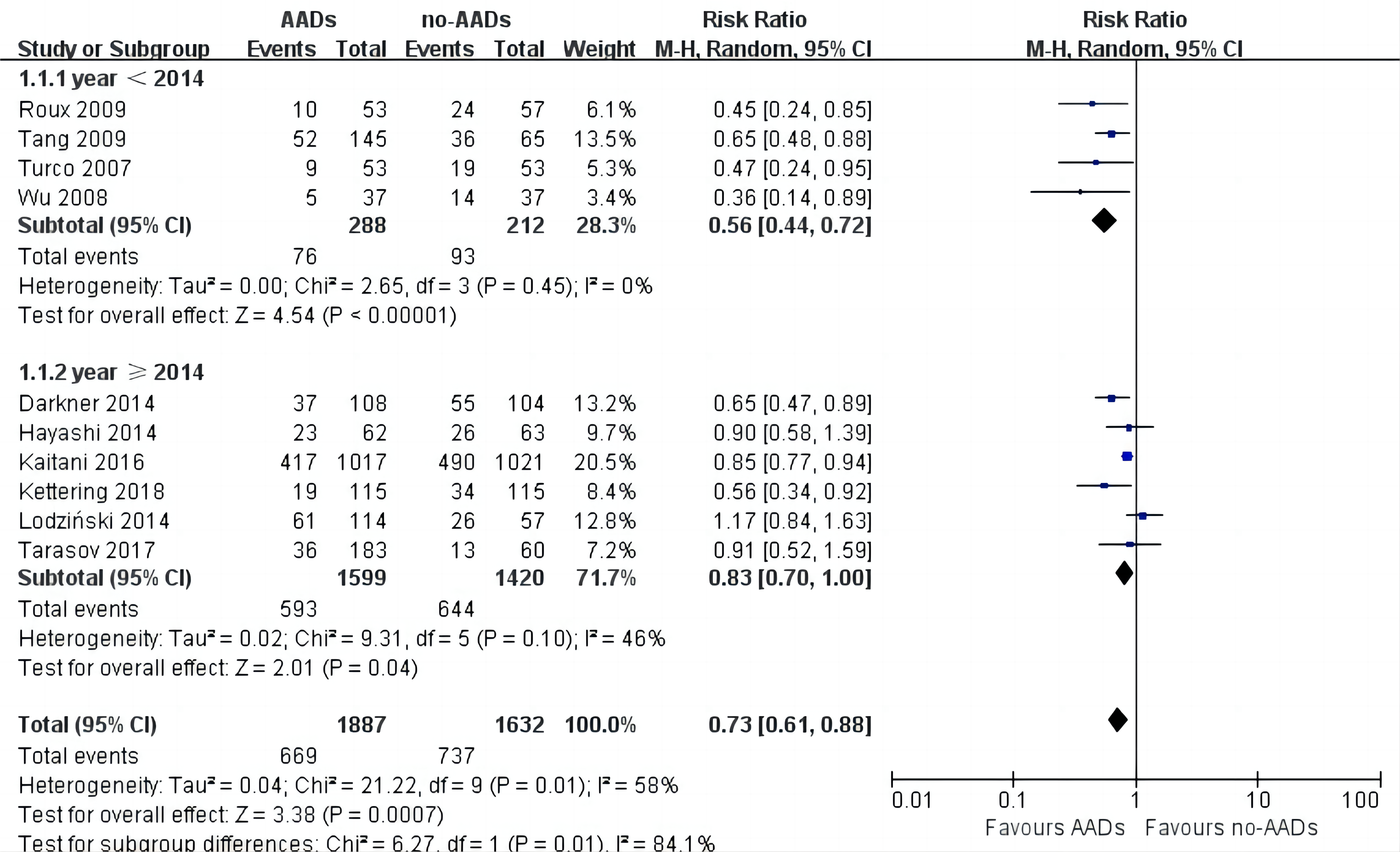

Supplement: Supplementary file 1 [file Image1.png]

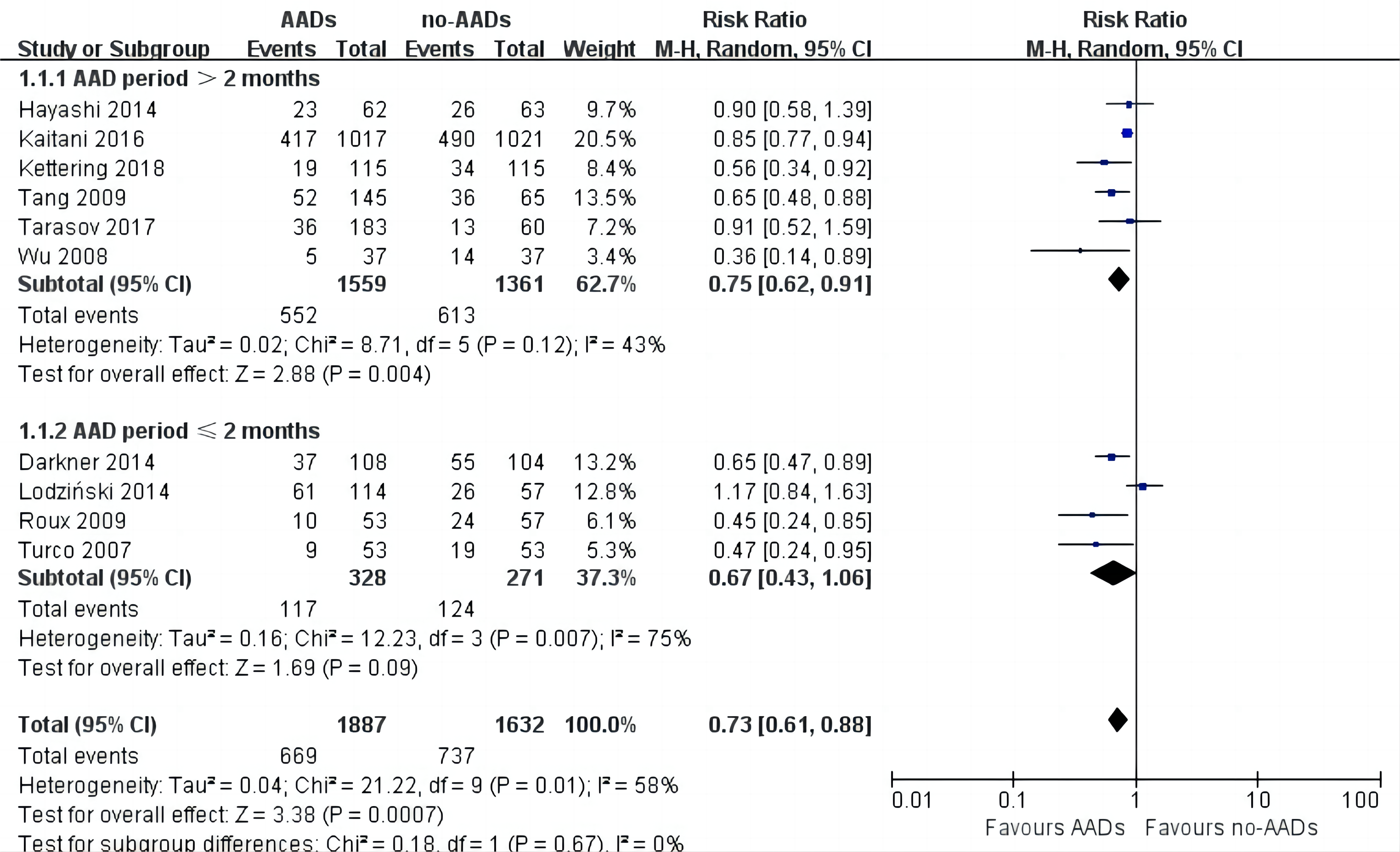

Supplement: Supplementary file 2 [file Image2.png]

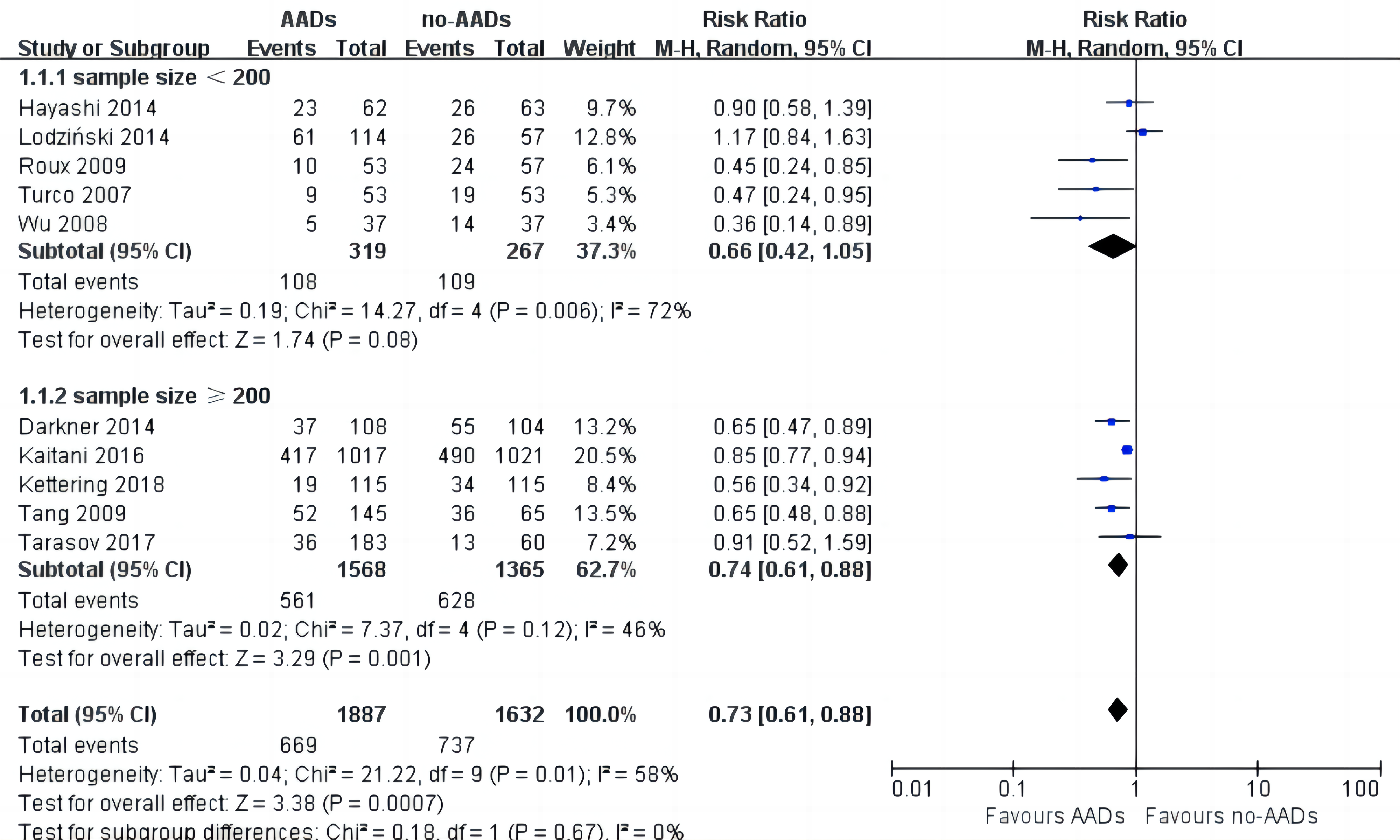

Supplement: Supplementary file 3 [file Image3.png]

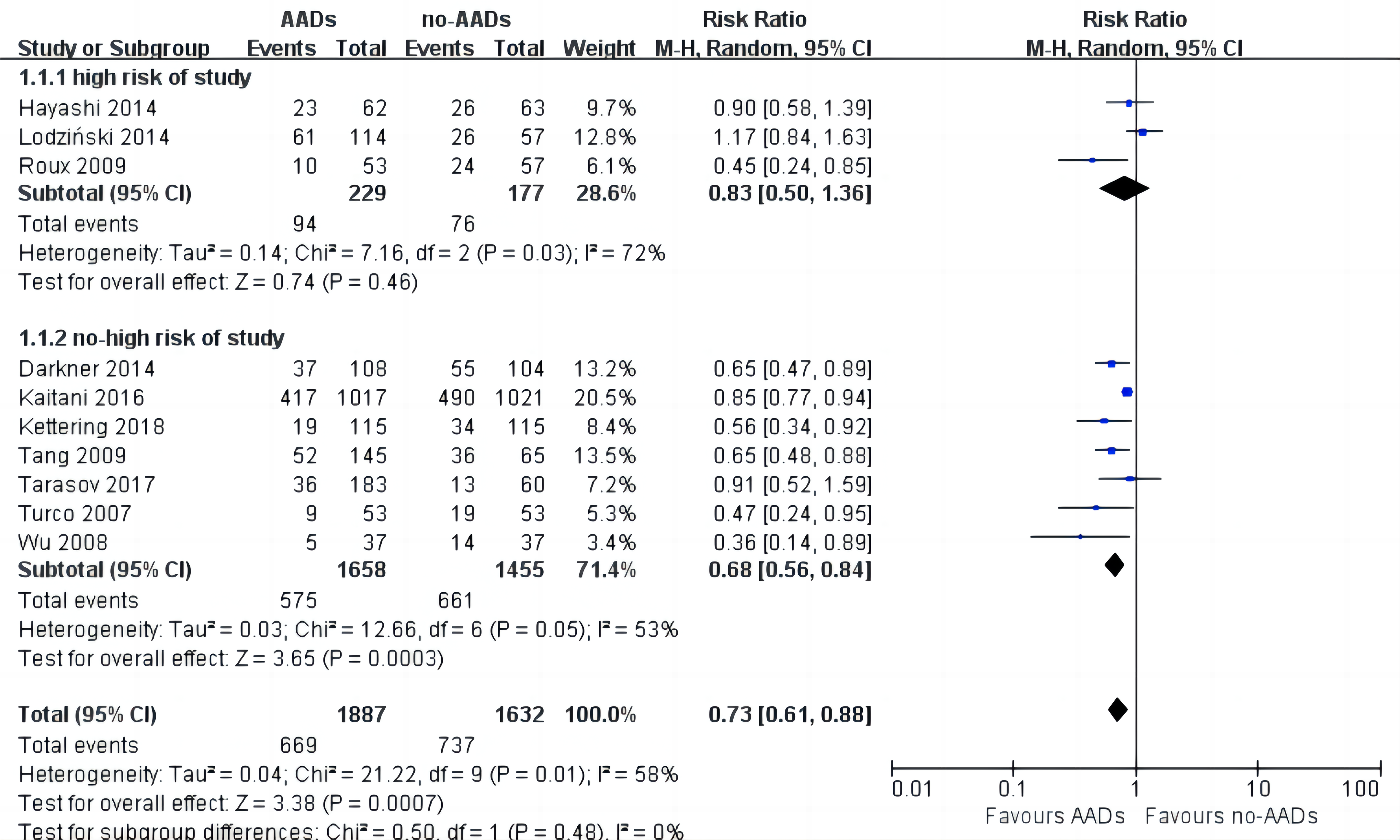

Supplement: Supplementary file 4 [file Image4.png]

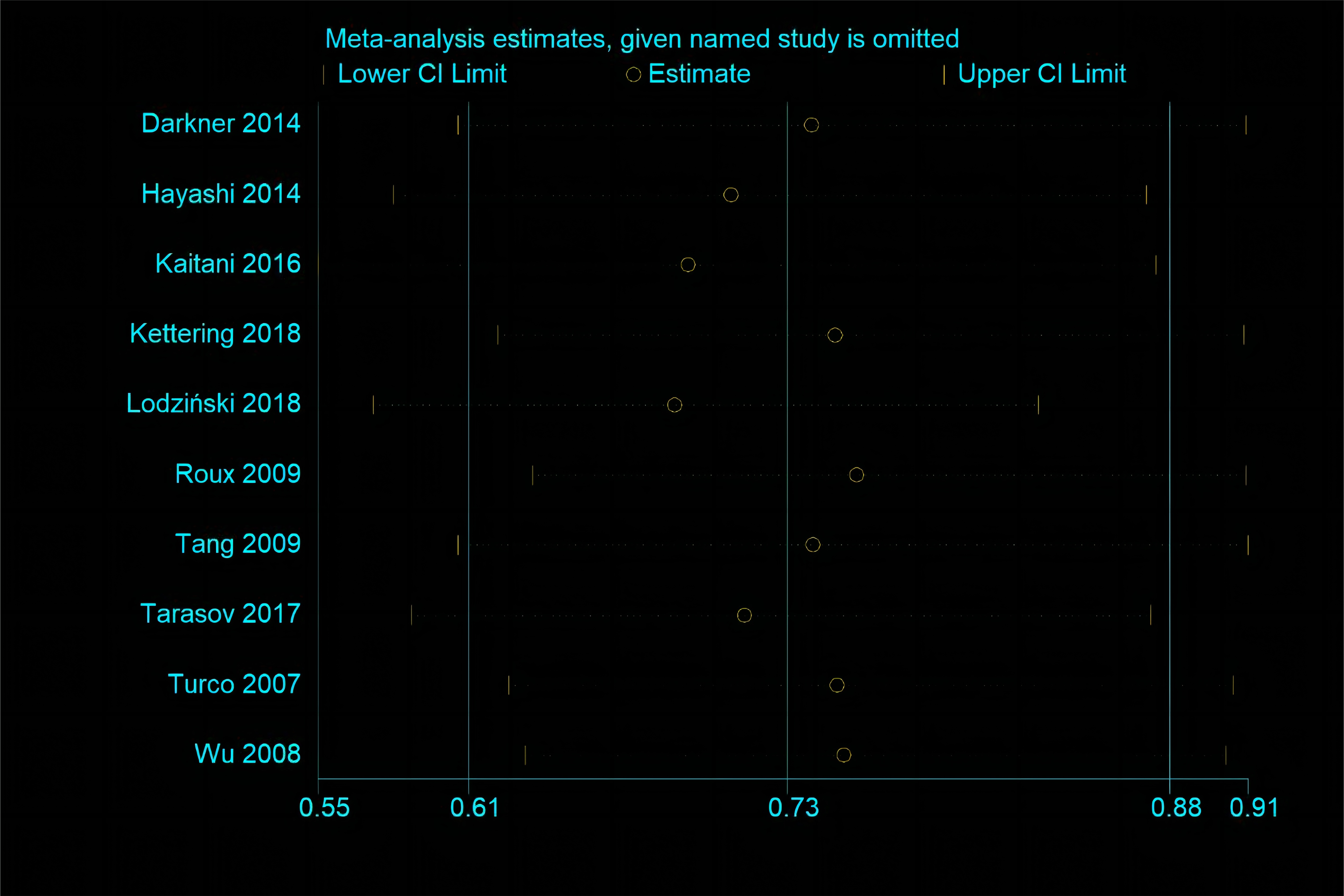

Supplement: Supplementary file 5 [file Image5.png]

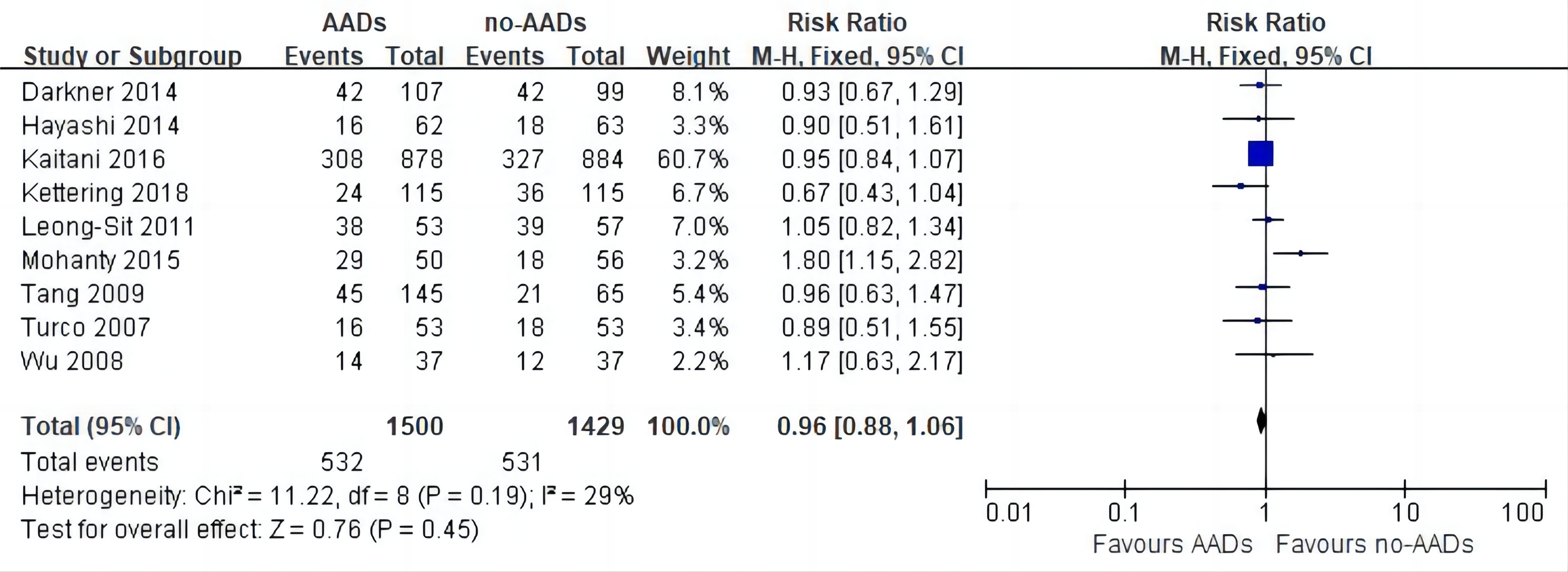

Supplement: Supplementary file 6 [file Image6.png]

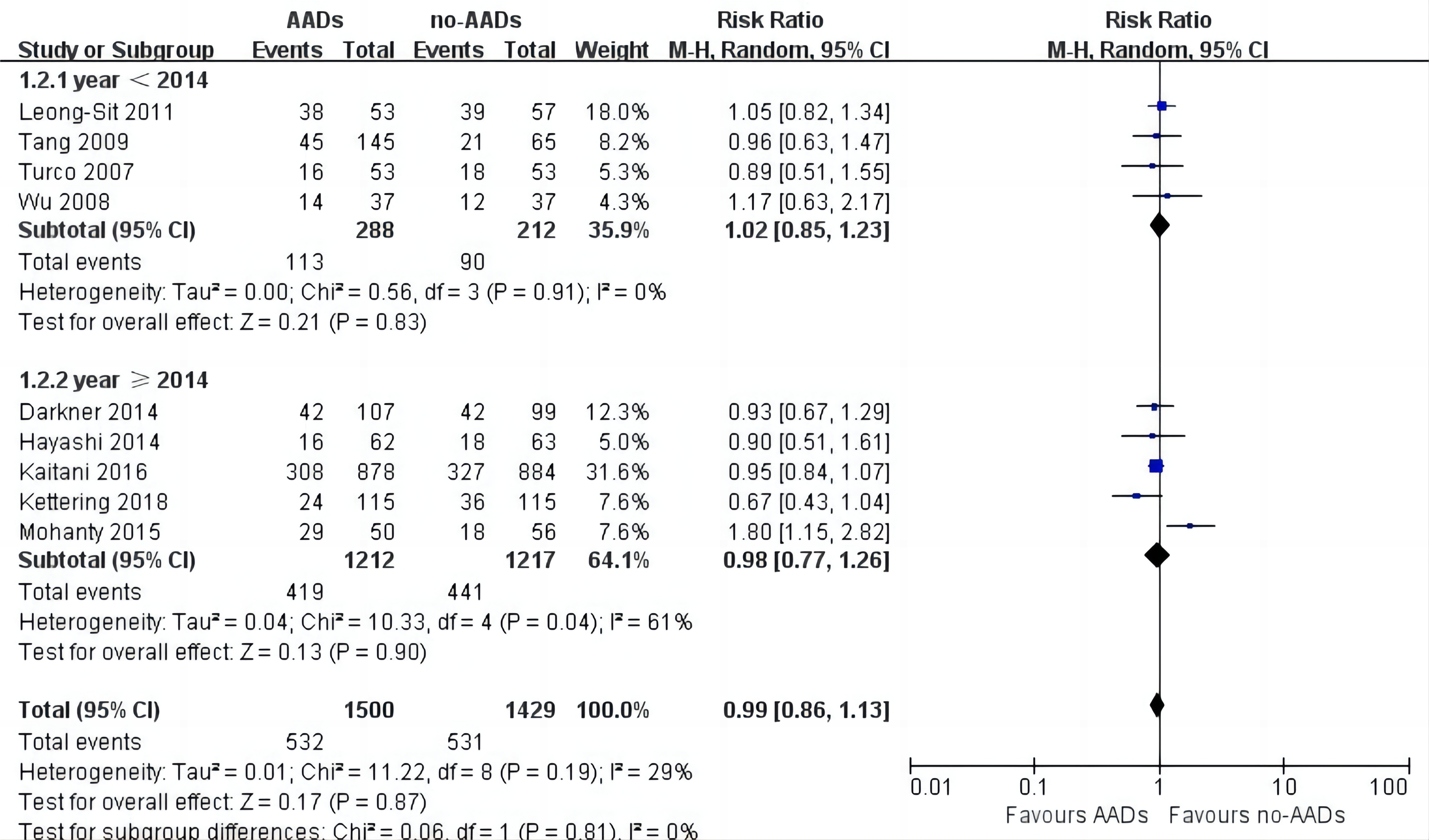

Supplement: Supplementary file 7 [file Image7.png]

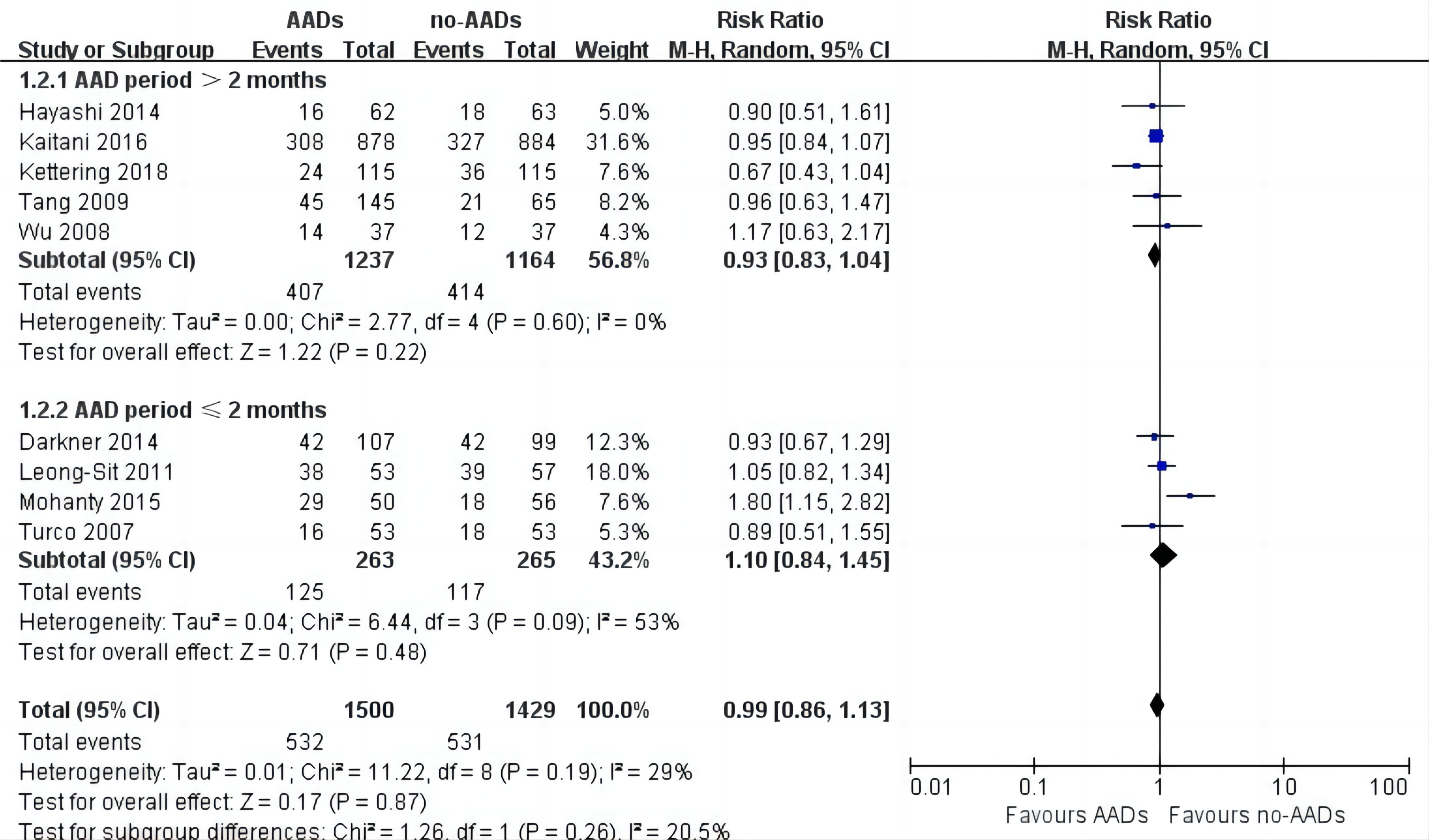

Supplement: Supplementary file 8 [file Image8.png]

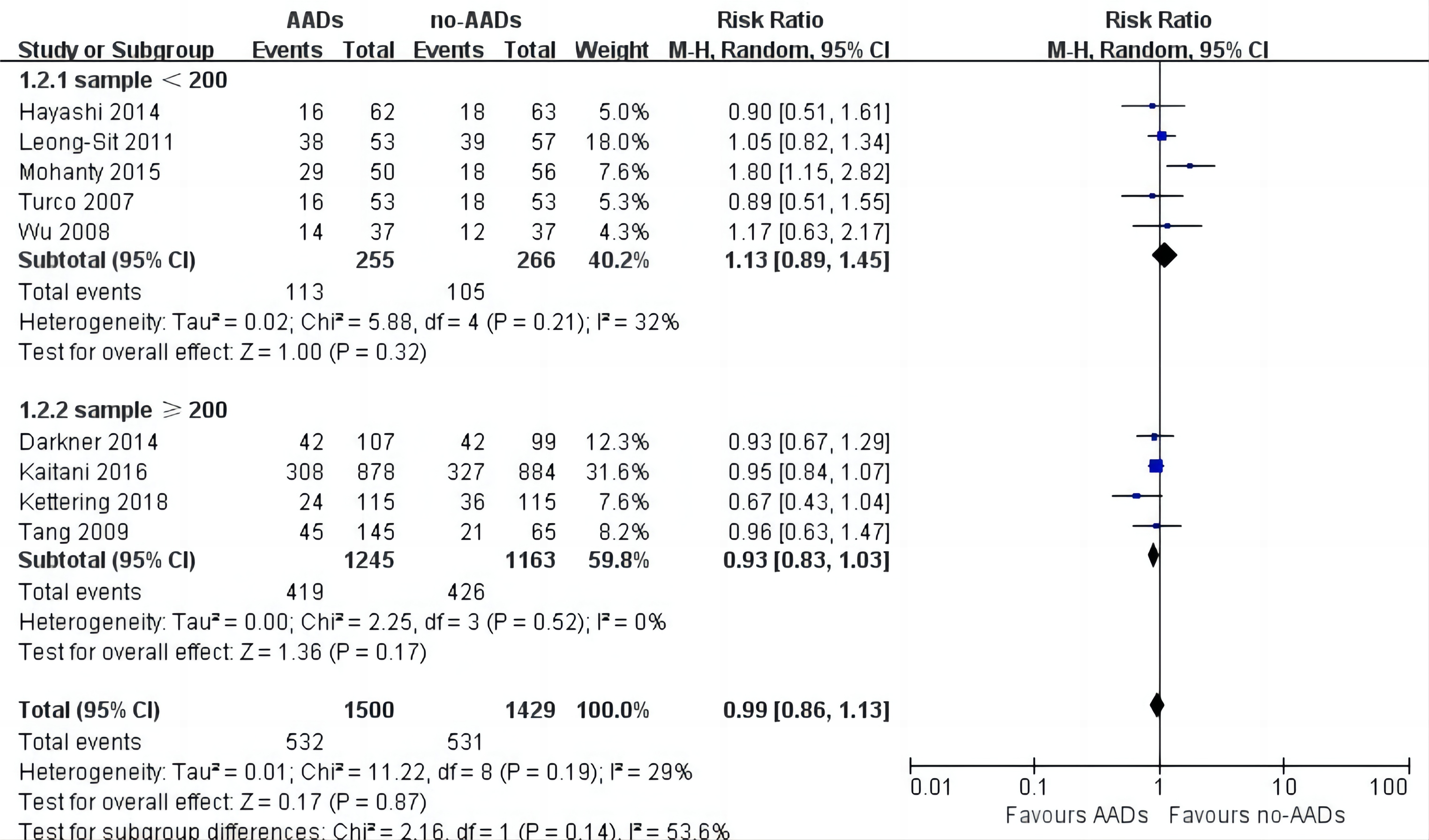

Supplement: Supplementary file 9 [file Image9.png]

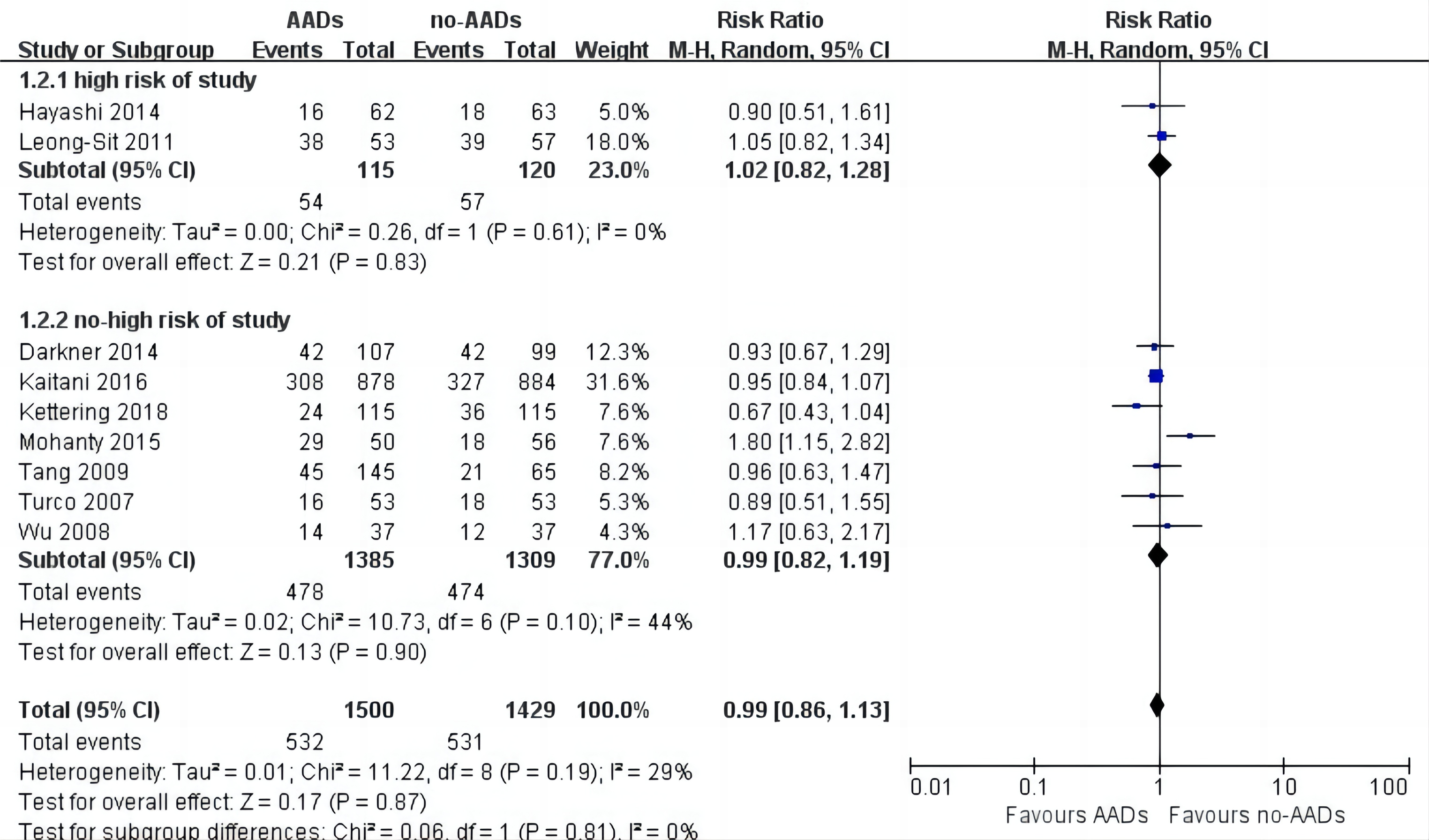

Supplement: Supplementary file 10 [file Image10.png]

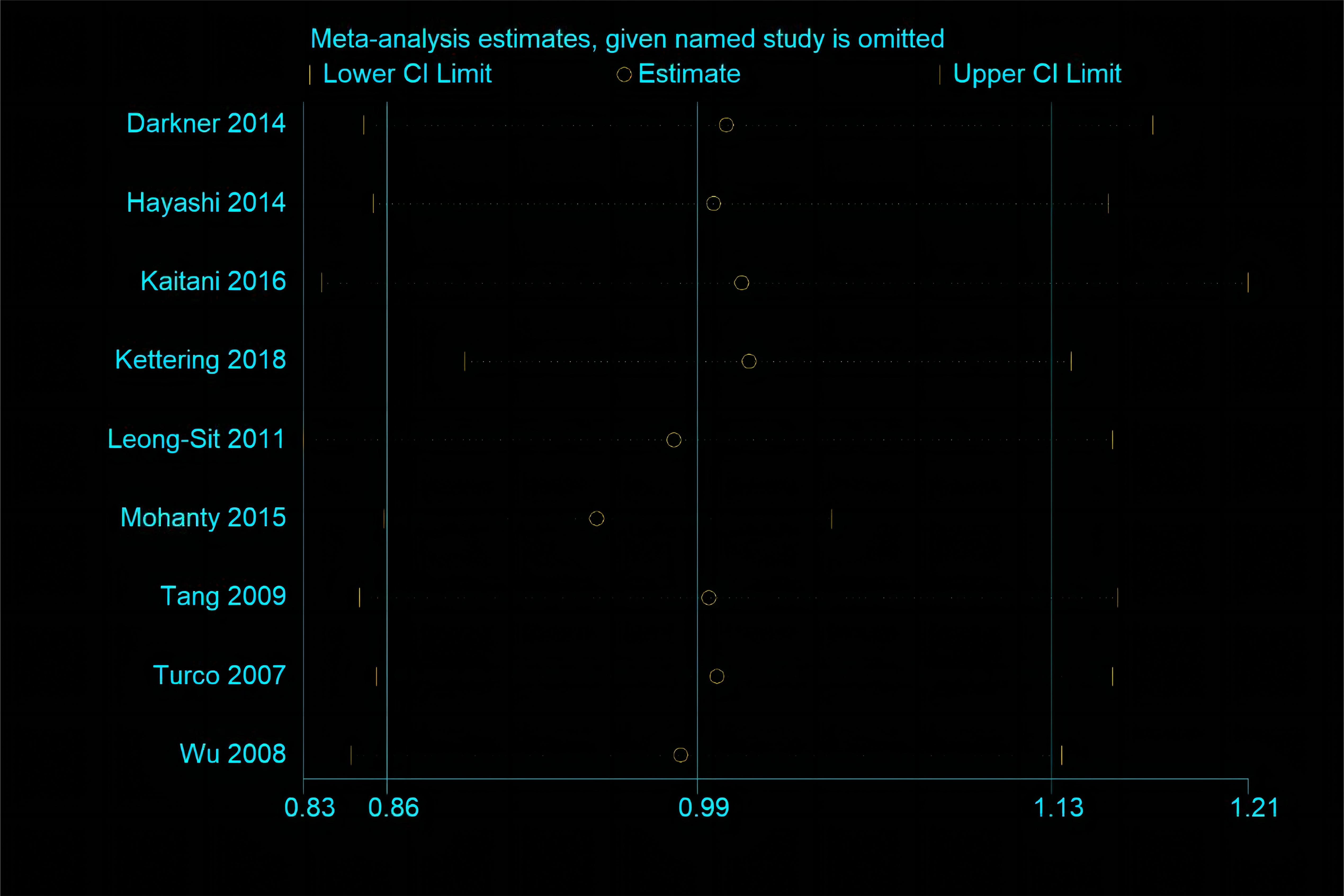

Supplement: Supplementary file 11 [file Image11.png]
